# Rethinking non-syndromic hearing loss and its mimics in the genomic era

**DOI:** 10.1038/s41431-024-01579-x

**Published:** 2024-03-06

**Authors:** Barbara Vona

**Affiliations:** 1 Institute of Human Genetics, University Medical Center Göttingen, 37073 Göttingen, Germany; 2 Institute for Auditory Neuroscience and InnerEarLab, University Medical Center Göttingen, 37075 Göttingen, Germany

**Keywords:** Diseases of the nervous system, Epidemiology, Genetic counselling

## Abstract

**Figure:**
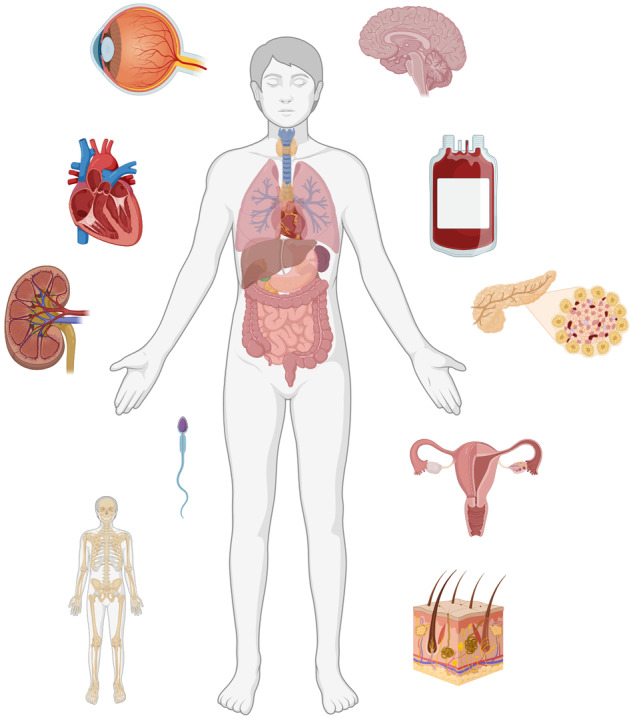
The many syndromes involving hearing loss.

The many syndromes involving hearing loss.

The sense of hearing plays a crucial role in everyday life, from influencing speech and language development in early childhood to reducing risk of social isolation, depression and cognitive decline in the elderly. The causes of hearing loss are numerous, although genetic causes are thought to be implicated in up to 80% of congenital diagnoses (reviewed in ref. [1]). The remarkable complexity of the auditory system is mirrored in its extensive genetic heterogeneity, with deleterious variants in hundreds of genes already associated with hearing loss and many more awaiting discovery. Clinically, hearing loss is arranged into two categories with syndromic or non-syndromic designations. Understanding potential syndromic involvement is crucial, as syndromic hearing loss comprises approximately 30% of genetic diagnoses in pediatric cases. This viewpoint aims to raise awareness of non-syndromic mimics and emphasizes the importance of a molecular genetic diagnosis in broadening understanding of the natural history of disease.

Before the widespread availability of molecular diagnostic testing, physicians would typically conduct a battery of diagnostic tests in newly diagnosed patients with clinically confirmed hearing loss. These tests included serology to assess infection, ophthalmology examinations, urinalysis, renal ultrasound, and electrocardiogram, among others, to identify common or potentially life-threatening syndromes [2]. However, with the introduction of molecular genetic testing, early diagnosis of syndromes, including those falling into a third unofficial category known as “non-syndromic mimics,” has become possible and stands as an important complication to the syndromic/non-syndromic hearing loss dichotomy that has long guided dialog in genetic counseling. Non-syndromic mimics initially present as either isolated hearing loss with delayed onset of other clinical features or as syndromes with mild or even sub-clinical manifestations initially overlooked during pre-testing counselling. As routine application of comprehensive genetic testing is still a historically new addition to the medical care of patients, the appreciation of possible outliers of expressivity, penetrance, severity, progression and sequential order of other phenotypes is often limited. As more patients undergo molecular diagnostic testing, there seem to be increasing reports of unusual clinical findings that merit publication – many of such articles are published in the European Journal of Human Genetics.

A significant challenge in high-throughput sequencing diagnostics is the identification of a substantial number of children and young adults who are clinically diagnosed with non-syndromic hearing loss who harbor variants in genes linked to syndromes. Adding to this is the growing number of genes that are implicated in both syndromic and non-syndromic hearing loss. So far, nearly 80 genes have been implicated as being non-syndromic mimics, with the list of genes continuously growing (Table 1). More syndromes than previously appreciated may present as non-syndromic hearing loss, potentially leading to the underdiagnosis of syndromic hearing loss. In practical terms, non-syndromic mimics appear as one of two possible scenarios.

**Table 1:** Syndromic genes that may mimic non-syndromic hearing loss.

Gene	Inheritance	Syndrome	Gene	Inheritance	Syndrome
Vision Impairment					
MYO7A*	AR	Usher 1B	*CLRN1*	AR	Usher 3A
CDH23*	AR	Usher 1D	*ARSG*	AR	Usher 4
PCDH15*	AR	Usher 1F	*PSIP1*	AD	Deafness and optic neuropathy
USH1G*	AR	Usher 1G	*SLITRK6*	AR	Deafness and myopia
WHRN*	AR	Usher 2D	*CEP250*	AR	Cone-rod dystrophy and hearing loss 2
*ADGRV1*	AR	Usher 2C	*CEP78*	AR	Cone-rod dystrophy and hearing loss
*USH2A*	AR	Usher 2A			
Renal Dysfunction					
*COL4A5*	X-linked	Alport 1	*COL4A3*	AR, AD	Alport 2; Alport 3
*COL4A4*	AR	Alport 2	BSND*	AR	Bartter 4A
Cardiac Dysfunction					
*KCNQ1*	AR, AD	Jervell and Lange-Nielsen; Long QT 1	*KCNE1*	AR, AD	Jervell and Lange-Nielsen; Long QT 5
Female Infertility					
*HSD17B4*	AR	Perrault 1; D-bifunctional protein deficiency	*SGO2*	AR	Perrault
*HARS2*	AR	Perrault 2	*PRORP*	AR	Perrault
*CLPP*	AR	Perrault 3	*RMND1*	AR	Perrault
*LARS2*	AR	Perrault 4	*GGPS1*	AR	Perrault
*TWNK*	AR	Perrault 5	*PEX6*	AR	Perrault
*ERAL1*	AR	Perrault 6	*TFAM*	AR	Perrault
Male Infertility					
*STRC-CATSPER2* Contiguous Deletion	AR	Deafness infertility syndrome	*POLR2C*	AR	Hearing loss and male infertility
CDC14A*	AR	DFNB32, with or without immotile sperm			
Connective Tissue Disorders (eye, bone)					
*COL2A1*	AD	Stickler 1; Kniest dysplasia	*COL9A2*	AR AD	Stickler 5; Epiphyseal dysplasia, multiple, 2
COL11A1*	AD AR	Stickler 2; Fibrochondrogenesis 1	*COL9A3*	AD	Epiphyseal dysplasia, multiple, 3, with or without myopathy
COL11A2*	AR, AD	Fibrochondrogenesis 2	*BMP4*	AD	Stickler with renal dysplasia
*COL9A1*	AR, AD	Stickler 4, Epiphyseal dysplasia, multiple, 6			
Waardenburg Syndrome					
*PAX3*	AD AR/AD AD	Waardenburg 1; Waardenburg 3; Craniofacial-deafness-hand	*EDNRB*	AR/AD AR	Waardenburg 4A; ABCD
*MITF*	AD AD AR	Waardenburg 2A; Tietz-albinism-deafness; COMMAD	*EDN3*	AR/AD	Waardenburg 4B
*SNAI2*	AR	Waardenburg 2D	*SOX10*	AD AD	Waardenburg 4C; Waardenburg 2E, with/without neurologic involvement
Heimler Syndrome					
*PEX1*	AR	Heimler 1; Peroxisome biogenesis disorder 1A and 1B	*PEX26*	AR	Heimler; Peroxisome biogenesis disorder 7A and 7B
*PEX6*	AR, AD	Heimler 2; Peroxisome biogenesis disorder 4A and 4B			
Branchio-otic					
*EYA1*	AD	Branchiootic 1	SIX1*	AD	Branchiootic 3
Pendred					
SLC26A4*	AR	Enlarged vestibular aqueduct Pendred	*KCNJ10*	AR	Enlarged vestibular aqueduct, digenic (Pendred); SESAME
*FOXI1*	AR	Enlarged vestibular aqueduct (Pendred)			
Wolfram Syndrome					
*CISD2*	AR	Wolfram 2	*WFS1*	AR AD	Wolfram 1 Wolfram-like
Heterogeneous Syndromes					
*ABHD12*	AR	Polyneuropathy, hearing loss, ataxia, retinitis pigmentosa, and cataract	*KARS1*	AR	Congenital deafness and adult-onset progressive leukoencephalopathy
ACTG1*	AD	Baraitser-Winter 2	*KMT2C*	AD	Kleefstra 2
*CHD7*	AD, AD	CHARGE; Hypogonadotropic hypogonadism 5 with/without anosmia	*KMT2D*	AD	Kabuki 1
DIAPH1*	AD	Thrombocytopenia	MYH9*	AD	Macrothrombocytopenia and granulocyte inclusions with or without nephritis
*FGF3*	AR	Congenital deafness with inner ear agenesis, microtia, and microdontia	*PHYH*	AR	Refsum
*FGFR3*	AD	LADD 2	*PTPN11*	AD	LEOPARD 1; Noonan 1
*FITM2*	AR	Deafness and dystonia (Siddique)	*RAI1*	AD	Smith-Magenis
*GATA3*	AD	Hypoparathyroidism, sensorineural deafness, and renal dysplasia	*SERPINF1*	AR	Osteogenesis imperfecta VI
*GPRASP2*	X-linked	Hearing loss with inner ear abnormalities and facial dysmorphism	*TFAP2A*	AD	Branchiooculofacial
*HARS1*	AD	Charcot-Marie-Tooth, axonal, 2 W			

An asterisk (*) designates an association with both non-syndromic and syndromic hearing loss.

## Scenario 1: The syndrome is yet to manifest

This first scenario is best illustrated by conditions such as Usher syndrome, where the initial presentation involves congenital or early onset hearing impairment, followed years or even decades later by visual impairment (retinopathy). If vestibular deficits are present, they are usually not obvious in infancy. Although initially appearing as non-syndromic hearing loss, the gradual progression to low vision or blindness underscores the importance of early education and preventative measures. In such instances, the diagnosis of a syndrome without prior indicators also has profound implications for both patients and their families. For example, an early diagnosis of Usher syndrome would educate the family to avoid relying on sign language as a means of communication due to poor prognosis of vision loss. Other syndromes that may be encountered include goitre with Pendred syndrome, the numerous syndromes associated with male (e.g., deafness infertility syndrome) or female infertility (e.g., Perrault syndrome) in pre-pubertal children, or sudden cardiac death with Jervell and Lange-Nielsen syndrome, with the latter case having life-threatening consequences.

## Scenario 2: The syndrome presents mildly and goes unnoticed

In other instances, the indicators of a syndrome may already be present but in a mild or atypical manner, resulting in their oversight. In such examples, syndromes are clinically identified through retrospective evaluation following molecular genetic testing. Enhancing diagnostic accuracy can occasionally be achieved through a thorough dysmorphology assessment, which can reveal subtle dysmorphisms. As an example, the identification of dystopia canthorum in Waardenburg Syndrome type I through eye measurements illustrates the importance of a meticulous physical examination in uncovering syndromic features that may not be immediately evident. Another example pertains to missed abnormal branchial arches and kidneys in patients with branchio-oto-renal syndrome, where hearing loss typically starts at birth. In many cases, the degree of subtlety of the syndromic findings does not merit a clinical diagnosis or meet diagnostic criteria of the associated syndrome [3–5]. The broad spectrum of presentation and sheer number of hearing loss syndromes makes the probability of encountering such a scenario in clinical practice a possibility.

An early diagnosis of a non-syndromic mimic was unattainable before advanced molecular genetic testing. In fact, it was not until the widespread adoption of such testing that the term “non-syndromic mimic” emerged and only recently has it been possible to grasp an appreciation of its prevalence. However, estimating the occurrence of non-syndromic mimics continues to prove challenging, often relying on the recognition of healthcare professionals and a progressively refined understanding of the natural progression of such syndromes. Identifying genes that mimic non-syndromic hearing loss remains a hurdle. Nevertheless, it is crucial to raise awareness and the growing volume of reports detailing unusual natural progressions. Early identification of syndromes remains imperative, enabling prompt referral of affected individuals to specialists, facilitating tailored interventions, and enhancing prognostic accuracy.
